# The effect of spacers in dual drug-polymer conjugates toward combination therapeutic efficacy

**DOI:** 10.1038/s41598-021-01550-6

**Published:** 2021-11-11

**Authors:** Juan Xu, Mengdi Ma, Jean Felix Mukerabigwi, Shiying Luo, Yuannian Zhang, Yu Cao, Lifeng Ning

**Affiliations:** 1 National Research Institute for Family Planning, Beijing, 100081 People’s Republic of China; 2 Key Laboratory of Pesticide and Chemical Biology of Ministry of Education, College of Chemistry, Central China Normal University, Wuhan, 430079 People’s Republic of China; 3 Department of Chemistry, College of Science and Technology, University of Rwanda, P.O Box: 3900, Kigali, Rwanda

**Keywords:** Cancer, Drug discovery, Oncology

## Abstract

Recently, a great effort has been made to perfect the therapeutic effect of solid tumor, from single-agent therapy to combined therapy and many other polymer-drug conjugations with dual or more anticancer agents due to their promising synergistic effect and higher drug level accumulation towards tumor tissues. Different polymer-drug spacers present diverse therapeutic efficacy, therefore, finding an appropriate spacer is desirable. In this study, dual drugs that are doxorubicin (DOX) and mitomycin C (MMC) were conjugated onto a polymer carrier (xyloglucan) via various peptide or amide bonds, and a series of polymers drug conjugates were synthesized with different spacers and their effect on tumor treatment efficacy was studied both in vitro and in vivo. The result shows that the synergistic effect is better when using different linker to conjugate different drugs rather than using the same spacer to conjugate different drugs on the carrier. Particularly, the finding of this works suggested that, using peptide bond for MMC and amide bond for DOX to conjugate dual drugs onto single XG carrier could improve therapeutic effect and synergy effect. Therefore, in polymer-pharmaceutical formulations, the use of different spacers to optimize the design of existing drugs to enhance therapeutic effects is a promising strategy.

## Introduction

Treatment of tumor has seen great obstacle, despite the increasing number of researches focusing on this concern and a large number of therapeutic agents applied in this medical domain. In the treatment of solid tumors by traditional chemotherapy, its efficacy is related to the achievable drug concentration in the tumor, and the toxicity of chemotherapeutic agents. The continuous development of polymer-based diagnostic and therapeutic methods to treat human diseases is a research area that has been widely expanding. Consequently, several drugs were reported and progressed to clinical application^1^. Currently, carriers such as liposomes, various micelles nanoparticles, gene, and protein are well developed and extensively studied^2–4^. In addition, polymer conjugates as vehicles to deliver various drug at the pathological site have also been studied for clinical medical research, to achieve enhanced therapeutic effect^5–7^. Xyloglucan (XG) is a kind of natural polysaccharide, which has been extensively studied and used in various biomedical applications due to its excellent biocompatibility and non-toxic which make it an appropriate material for drug delivery system^8–10^. It has interesting functional groups that can be used to conjugate two or more drugs for combination therapeutic applications. More interestingly, XG showed to have some anti-bacterial properties and anti-cancer activities which make it more applicable in many medical devices.

Combination therapy offers huge advantages for cancer treatment compared to monotherapy^11^. Among the strategies used to achieve combinational therapy, the conjugation of therapeutic drugs to the polymer via various linkers also known as spacers showed promising potential to achieve enhanced therapeutic effect in cancer treatment. Despite the fact that different properties such as drug ratio, and polymer size, etc., that may influence the efficacy of combination therapy, it is widely known that the type of spacer used to conjugate the anticancer drug to the polymer can also significantly plays a great role to successfully release the parent drug at the desirable pathological site preferably in controllable manner^12,13^. In this scenario, various spacers that can respond to various endogenous and exogenous stimulus to achieve on-demand release of the parent drugs at the treatment sites have been widely reported. For example, a variety of spacers containing ester bonds, amide bonds, and enzymatically cleavable peptide bonds have been the most frequently used to attach anticancer agents such as mitomycin C (MMC), doxorubicin (DOX), paclitaxel (PTX), and camptothecin (CPT), etc.^14–16^. In addition, some pH-sensitive bonds like acetal and hydrazone bonds which can be hydrolyzed in the mild acidic environment of the endosomal and lysosomal compartments have been reported^15^. However, given the fact that different spacers are expected to have different properties that can strictly influence the treatment efficacy, finding an appropriate spacer is desirable for combination therapy.

This research is focusing on studying the cleavage of two different spacers that are amide bond and peptide bond between carrier and anticancer agents and their effect to achieve enhanced treatment efficacy for cancer. Briefly, doxorubicin (DOX) and mitomycin C (MMC) were conjugated onto the xyloglucan (XG) polymeric carrier through two methods *i.e.,* through peptide bond or amide bond. XG conjugated doxorubicin/mitomycin C derivatives via peptide/amide were synthesized and characterized by ^1^H-NMR spectroscopy. In vitro drug release profile was measured by using a clonogenic assay. Moreover, the therapeutic effect on drug resistance liver hepatocellular carcinoma tumor cell line (HepG2/DR) was evaluated both in vitro and in vivo. Interestingly, unlike using the same spacer to attach different drugs to carriers in combination therapy, the results of this research revealed that using different spacers for the different drugs could achieve a better therapeutic effect.

## Experimental

### Materials

XG prepared from tamarind seed powder was procured from TCI (Shanghai, China). DOX and MMC were purchased from Chuangcheng Pharmaceutical Ltd., (Wuhan, China). N-t-Boc-glycyl-L-leucyl-glycine N-hydroxysuccinimideester (Boc-Gly-Leu-Gly-OSu) and 12-(boc-amino) aminododecanoic acid n-succinimidyl este (BOC-ADA-OSU) were supplied by GL Biochem Ltd., (Shanghai, China). 3-(4,5-Dimethylthiazol-2-yl)-2,5-diphenyltetrazolium bromide (MTT) and collagenase IV were obtained from the Sigma-Aldrich Co, Ltd., USA. Human hepatocellular carcinoma cell line (HepG2) was obtained from China Center for Type Culture Collection (Wuhan, China). 5 weeks-old female BALB/c nude mice were purchased from Shanghai Institute of Materia Medica, Chinese Academy of Sciences (Shanghai, China). Unless otherwise stated, all chemicals used are of analytical grade.

### Preparation of DOX/MMC-peptide and DOX/MMC-CONH derivatives

1 g of DOX (1.84 mmol) and 0.75 g of Boc-Gly-Leu-Gly-OSu (1.70 mmol) were dissolved into dry DMF and 0.4 g (0.25 mmol) of diethyl phosphoryl cyanide (DEPC) was added under a moderate stirring. After stirring for 0.5 h, 0.3 mL of triethylamine (TEA) was added. After overnight reaction in the dark at room temperature, the solvent was evaporated in vacuum and ethyl acetate was added to dissolve the dry residue. The reaction mixture was extracted with a 10% citric acid solution (3 × 5 mL) and saturated sodium bicarbonate (3 × 5 mL). The organic layer was isolated and the water layer extracted with ethyl acetate (2 × 5 mL). Ethyl acetate extracts were evaporated to dryness in vacuum, and the residue was purified by column chromatography on silica. The selected fraction was dried over MgSO_4_. After removal of the solvent, the Boc-Gly-Leu-Gly-DOX derivative was finally obtained. 0.1 g of Boc-Gly-Leu-Gly-DOX was dissolved in 2 mL DMF, 0.2 mL of trifluoroacetic acid (TFA) was added. The reaction was conducted at room temperature for 1 h under stirring. In a vacuum environment, the solvent is evaporated. The residue was dissolved in 5 mL methanol, and the solution was filtered^17^. The Gly-Leu-Gly-DOX conjugate was finally obtained after evaporation of the solvent.

0.44 g of Boc-Gly-Leu-Gly-OSu (1 mmol) and 0.37 g of MMC (1.1 mmol) were dissolved in 20 mL DMF and 0.22 g of DEPC was added with stirring. Add 0.15 mL of TEA at 0 °C and stir for 0.5 h. After overnight reaction in the dark at room temperature, the solvent was evaporated under vacuum and ethyl acetate was added to dissolve the dry residue. The reaction mixture was extracted with a 10% citric acid solution (3 × 5 mL) and saturated sodium bicarbonate (3 × 5 mL). The organic layer was isolated and the water layer extracted with ethyl acetate (2 × 5 mL). Ethyl acetate extracts were evaporated to dryness in vacuum, and the residue was purified by column chromatography on silica (eluent: CHCl_3_/MeOH, 9/1). The selected fraction was dried over MgSO_4_. After removal of the solvent the Boc-Gly-Leu-Gly-MMC derivative was finally obtained as a blue solid.

0.1 g of Boc-Gly-Leu-Gly-MMC was dissolved in 2 mL of DMF, then 0.2 mL of TFA was added, and the reaction was further carried out with stirring at room temperature for 1 h. The solvent was evaporated *in vacuo*, then the residue was dissolved in 5 mL of methanol, and the solution was filtered. After evaporating the solvent again, Gly-Leu-Gly-MMC (1) conjugate was finally obtained.

Amino dodecanoic acid MMC/DOX derivatives (DOX/MMC-CONH) were prepared in the similar manner.

### Preparation of the XG-peptide-DOX/MMC and XG-CONH-DOX/MMC conjugates

XG (2 g, 0.025 mmol) and 4-dimethylaminopyridine (DMAP) (0.15 g, 1.2 mmol) were dissolved into 20 mL of DMSO/pyridine solution (vol. ratio 1/1). At 0 °C, 4-nitrophenyl chloroformate (0.9 g, 4.4 mmol) was added. Then, the reaction mixture was continuously stirred at room temperature for 4 h, and then subjected to precipitation treatment with absolute ethanol. A white precipitate was gained and washed repetitively with the same solvent. The XG-COO(C_6_H_4_)NO_2_ was finally dried in vacuum. The carbonate content was determined by UV analysis after activated XG hydrolysis in NaOH.

2 g of XG-COO(C_6_H_4_)NO_2_ (1.3 mmol reactive groups) and 2 g of Gly-Leu-Gly-DOX and 2 g of Gly-Leu-Gly-MMC (1.2 mmol) were dissolved in dry DMSO and then TEA (0.1 mL) was added. After 48 h of reaction in darkness, the conjugate was separated by precipitation in anhydrous ethanol. First, the product was washed, and then dried. Finally, with the preparative HPLC (Sephadex G25) with water as eluent and freeze-drying , the conjugate was purified^9^. The content of DOX and MMC in the conjugates was determined by UV analysis in water.

A series of XG-MMC/DOX conjugates loading dual drugs with different spacer were prepared with DOX/MMC-peptide and DOX/MMC-CONH derivatives.

### In vitro release of MMC and DOX from the conjugates

The study of drug release was carried out in phosphate buffer solution (PBS, pH 7.4) incubated with collagenase IV (0.3 mg/mL) at 37 °C with mild stirring. The XG-peptide-MMC/DOX (DOX/MMC-peptide), XG-CONH-MMC/DOX (DOX/MMC-CONH), DOX-CONH/MMC-peptide and DOX-peptide/MMC-CONH conjugates were individually immobilized into 10 mL dialyzing bag with molecular weight cutoff (MWCO) 3000 Da and subjected to dialysis against PBS (pH 7.4) at 37 °C The samples (dialysate) were collected in time dependent manner and immediately analyzed by a Shimadzu HPLC system composed of two pumps (LC-10Avp and LC-10AS) and an SPD-10Avp ultraviolet detector (Shimadzu Corporation, Japan) in reverse phase mode at different points of time. Using an Extend-C18 column (4.6 × 250 mm I.D., 5 μm), and the mobile phase used for the analysis was methanol–acetonitrile-phosphate buffer (pH 5.0, 0.2 M) (50:20: 30, v/v/v) and the flow rate was 0.5 mL/min. According to the predetermined standards for each drug, the amount of DOX in the solution was quantified at 245 nm wavelength, and the amount of MMC was determined at 360 nm wavelength.

### In vitro cytotoxicity assay

The DOX resistant HepG2 cell line (HepG2/DR) was developed by adding the increasing DOX concentration from 0.01 to 2 μg/mL in the period of 3 months. The selection of resistant cells was obtained by washing-off dead non-resistant cells. The drug resistance was maintained by culturing the cells with 1 μg/mL DOX^9^. The cytotoxicity of the conjugates was investigated against drug resistant human hepatoma cell line (HepG2/DR) with the 3-[4,5-dimethylthiazol-2-yl]-2,5-diphenytetrazolium (MTT) assay. HepG2/DR cells were seeded at a density of 1 × 10^4^ cells/well into 96-well culture plates at 37 ℃, and in a humidified environment containing 5% CO_2_, in 100 µL RPMI 1640 culture medium supplemented with 10% fetal bovine serum (FBS), penicillin and streptomycin (5%). Then different formulations as mentioned in Table 1 were used to treat the cells for 48 h. Thereafter Next, the solution was aspirated and replaced by 100 µL fresh medium followed by addition of MTT solution (20 µL, 5 mg/mL) and incubation of 4 h. Finally, the solution was replaced by adding 200 µL DMSO and placed on shaking bed for 15 min in dark before being put into microplate reader to measure the absorbance at the wavelength of 570 nm. Data was expressed by cell survival. The reversal of multi drug resistance (MDR) was measured by the half maximal inhibitory concentrations (IC_50_).

**Table 1:** List of abbreviations in formulation.

Formulation	Full name	Linker	Polymer quantity
XG	Xyloglucan	–	–
DOX	Doxorubicin	–	–
MMC	Mitomycin C	–	–
DOX-CONH	Doxorubicin conjugated macromolecule via amide bond	Amide bond	1
MMC-CONH	Doxorubicin conjugated macromolecule via amide bond	Amide bond	1
DOX-peptide	Doxorubicin conjugated macromolecule via peptide bond	Peptide bond	1
MMC-peptide	Mitomycin C conjugated macromolecule via peptide bond	Peptide bond	1
MMC/DOX- peptide	Mitomycin C conjugated macromolecule via peptide bond, and doxorubicin conjugated macromolecule via peptide bond	peptide bond	2
MMC/DOX-CONH	Mitomycin C conjugated macromolecule via amide bond, and doxorubicin conjugated macromolecule via amide bond	Amide bond	2
DOX-CONH/MMC-peptide	Doxorubicin conjugated macromolecule via amide bond, and mitomycin C conjugated macromolecule via peptide bond	Amide bond; peptide bond	2
DOX-peptide/MMC-CONH	Doxorubicin conjugated macromolecule via peptide bond, and mitomycin C conjugated macromolecule via amide bond	Peptide bond; amide bond	2
(Co)MMC/DOX- peptide	Mitomycin C and doxorubicin conjugated single macromolecule via peptide bond	Peptide bond	1
(Co)MMC/DOX-CONH	Mitomycin C and doxorubicin conjugated single macromolecule via amide bond	Amide bond	1
(Co)DOX-CONH/MMC-peptide	Doxorubicin conjugated single macromolecule via amide bond, and mitomycin C conjugated single macromolecule via peptide bond	Amide bond; peptide bond	1
(Co)DOX-peptide/MMC-CONH	Doxorubicin conjugated single macromolecule via peptide bond, and mitomycin C conjugated single macromolecule via amide bond	Peptide bond; amide bond	1

### Combination index (CI) determination

Combination index (CI), one of the simplest formalisms to describe synergy in combination drug therapy, is calculated according to the following formula:

$$ CI = \frac{{IC_{50} \left( A \right)_{pair} }}{{IC_{50} \left( {\text{A}} \right)}} + \frac{{IC_{50} \left( B \right)_{pair} }}{{IC_{50} \left( {\text{B}} \right)}} $$

where IC_50_(A)_pair_ and IC_50_(B)_pair_ are the half inhibitory concentration when drug given as an A-B pair; IC_50_(A) and IC_50_(B) are the half inhibitory concentration when drug A or B acts singly. The CI values lower than, equal to, and higher than 1 indicate synergism, additivity and antagonism, respectively.

### In vivo cytotoxicity of XG-peptide/amide-MMC/DOX conjugates against drug resistant HepG2/DR Cells in BALB/c nude mice

HepG2/DR drug resistant tumor model was subcutaneously implanted into 6 weeks-old female BALB/c nude mice. Three weeks later as the tumor size reached ~ 50 mm^3^, mice were randomly grouped into different treatment groups of 5 mice in each group, and XG-MMC/DOX conjugates or free DOX and MMC (equivalent dose of MMC and DOX = 25 μmol/Kg) suspended in PBS were administered via tail veins of mice every week for four doses (days 0, 7, 14, and 21). A major axis and a minor axis of tumors were measured with calipers. The volume of tumor was then determined. The number of long-term survivors and the survival time were also recorded^9^.

### Ethical Statement

All work implemented on animals was in accordance with the “Guidelines for the Care and Use of Laboratory Animals” published by the National Institute of Health (NIH Publication No. 85-23, revised 1985). This study was approved by the Ethics Committee of Central China Normal University (CCNU). All animal experiments are carried out complying with the regulations of "Regulations on the Administration of Experimental Animals" (second edition, revised in 2013) submitted by the National Science and Technology Commission. All animal experiments were conducted in compliance with the ARRIVE guidelines.

### Statistical analysis

Data with multiple comparisons were analyzed by Kruskal–Wallis one-way analysis of variance followed by Holm's Stepdown Bonferroni procedure. We used one-way analysis of variance (ANOVA) with the *Tukey* test or *t* test to compare the differences between the means of two groups at the same time point. Differences were considered to be statistically significant if *p* < 0.05^18^.

### Ethics approval and consent to participate

All animal experiments are carried out complying with the regulations of "Regulations on the Administration of Experimental Animals" (second edition, revised in 2013) submitted by the National Science and Technology Commission.

### Consent for publication

The manuscript is approved by all authors for publication.

## Results

### Synthesis and characterization of the XG-peptide-MMC/DOX and XG-CONH-MMC/DOX conjugates

**Figure 1:**
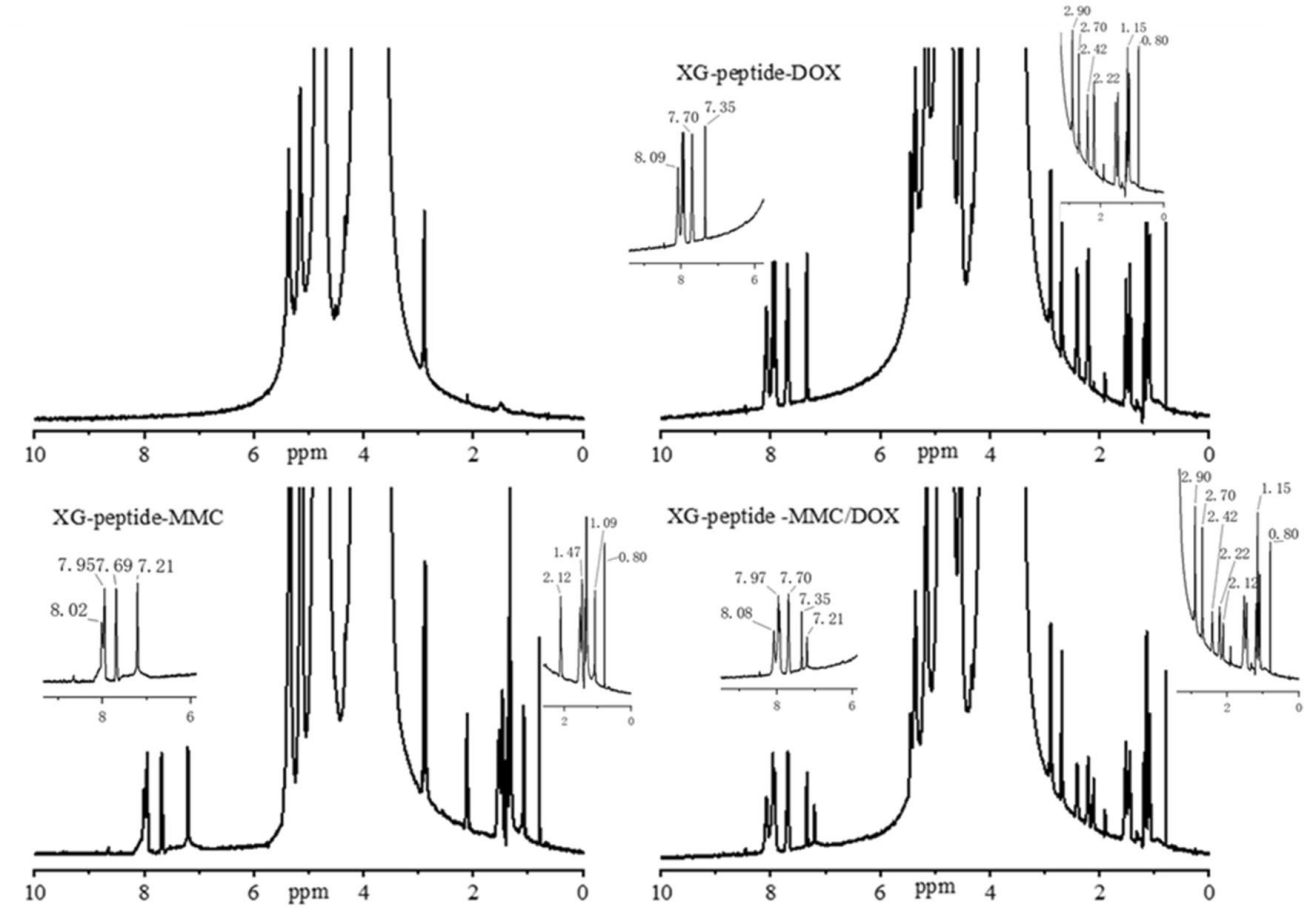
^1^H NMR spectroscopy of xyloglucan (XG), XG-peptide-DOX, XG-peptide-MMC, and XG-peptide -MMC/DOX conjugates.

**Figure 2:**
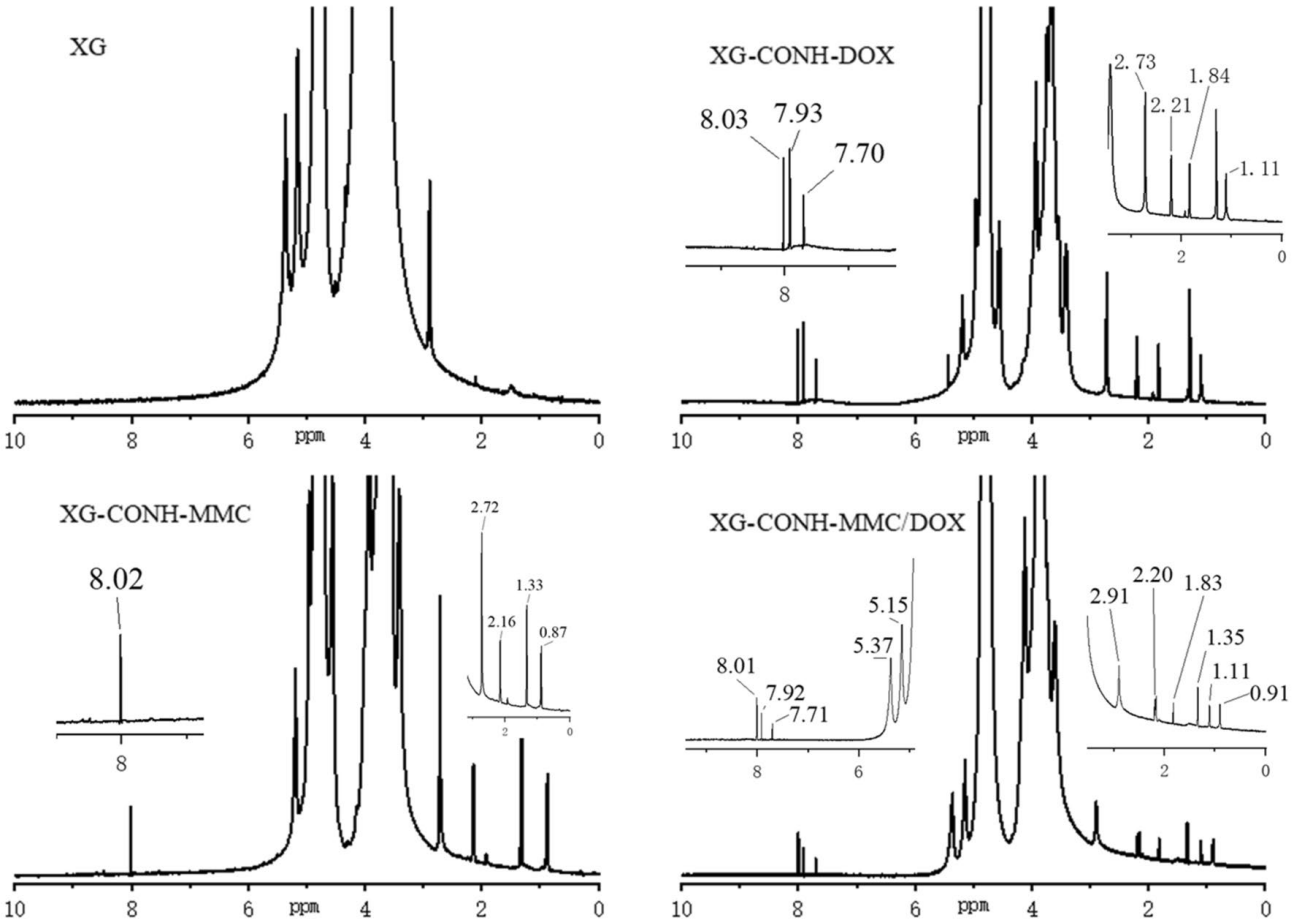
^1^H NMR spectroscopy of xyloglucan (XG), XG-CONH-DOX, XG-CONH-MMC, and XG-CONH -MMC/DOX conjugates.

**Figure 3:**
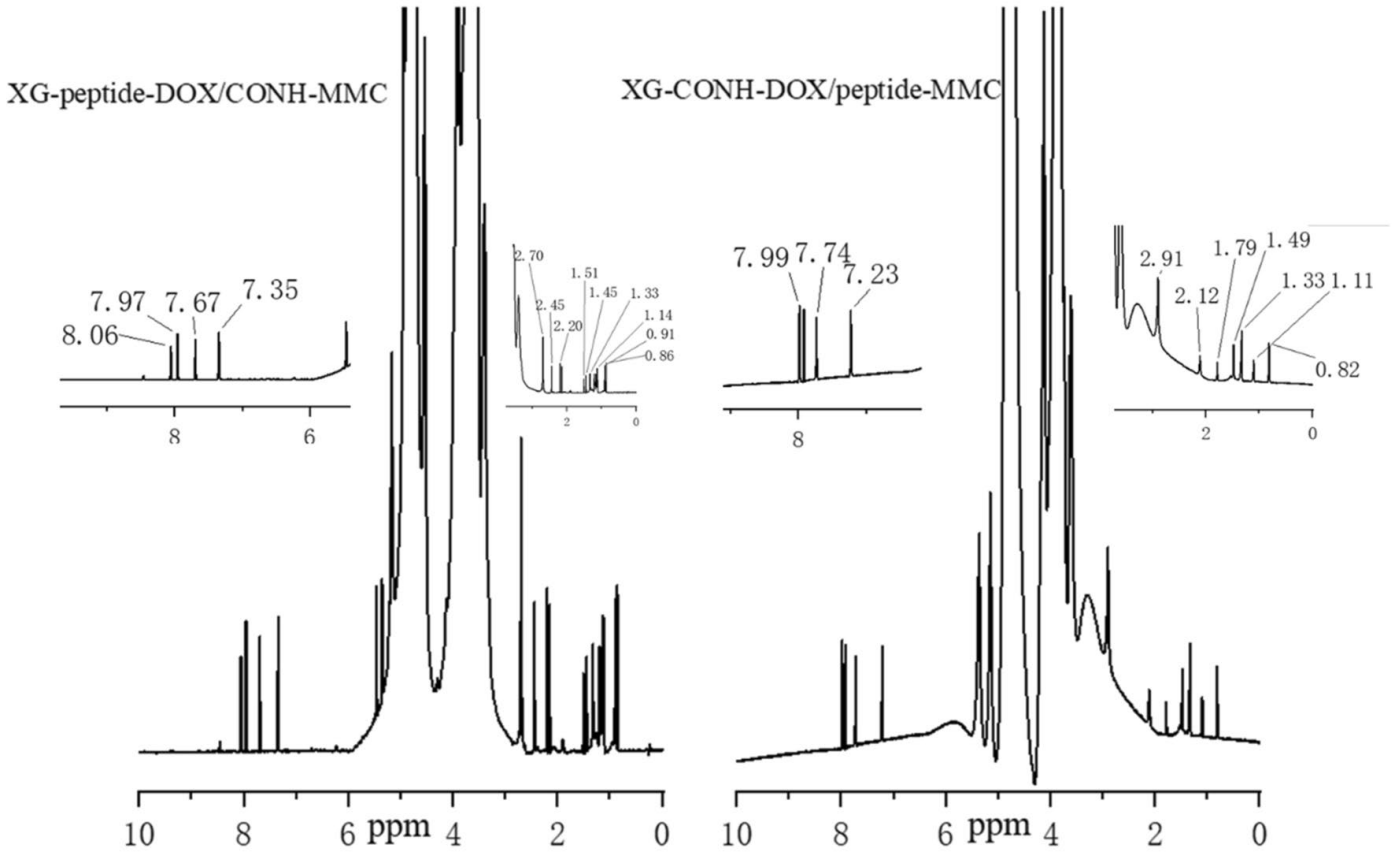
^1^H NMR spectroscopy of XG-peptide-DOX/CONH-MMC and XG-CONH-DOX/peptide-MMC conjugates.

In this study, by mixing polysaccharides with the 4- nitrophenyl chloroformate to activate XG, the prepared peptides and amide derivatives can be introduced into polymer carriers. The carbonyl and amino groups of MMC and DOX were suitable for grafting the drugs to carriers by amide bond and hydrazone bond, respectively. In Figs. 1, 2 and 3, the peaks from 7.7 to 8.0 ppm should be addressed to the absorption of the hydrogen of the benzene in the conjugated DOX. The peaks about 2.2 ppm, 2.4 ppm and 2.7 ppm might be assigned to the absorption of the hydrogen of the methylene in the conjugated DOX. The peaks about 7.2 ppm are referred to the absorption of the hydrogen of the amide group in the conjugated MMC. The peaks near 8.0 ppm should be attributed to the absorption of the hydrogen of the amide group between the carboxyl of peptides and the amino of quinone ring in MMC. The results of ^1^H NMR spectroscopy evidenced that MMC and DOX were successfully conjugated to the XG carrier.

### Size measurement

**Figure 4:**
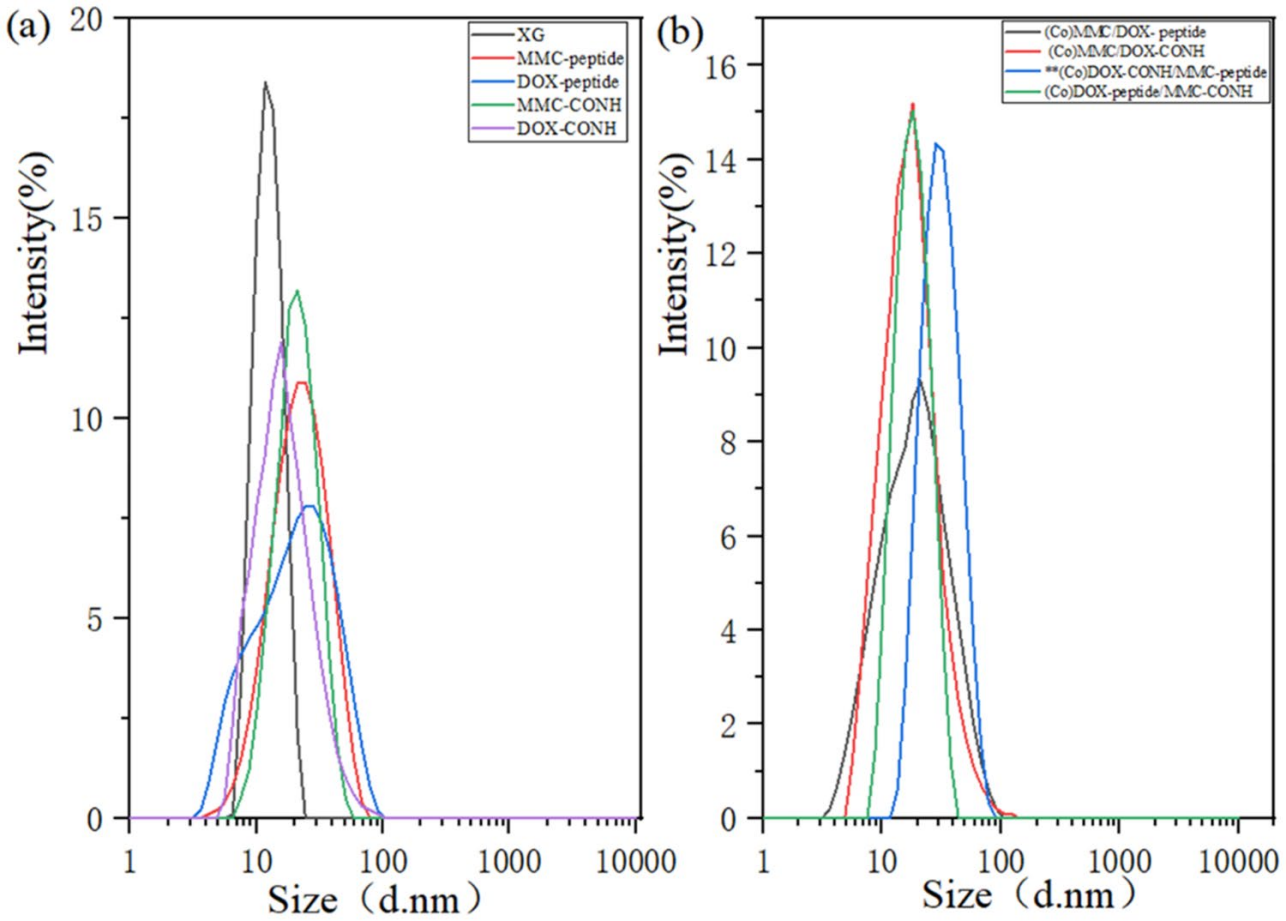
(**a**) Polymer size of XG, MMC-peptide, DOX-peptide, MMC-CONH,DOX-CONH (**b**) Polymer size of (Co)MMC/DOX-peptide, (Co)MMC/DOX-CONH, **(Co)DOX-CONH/MMC-peptide, (Co)DOX-peptide/MMC-CONH.

The sample particle size was measured through dynamic light scattering (DLS; Malvern Zetasizer nano-ZS90, Malvern Instruments Ltd, UK). As shown in Fig. 4, compared with XG, the molecular conformation changes and particle size slightly increases after modification of the XG polymer through conjugation of drug.

### Drug release from the XG-peptide-MMC/DOX and XG-CONH-MMC/DOX conjugates

**Figure 5:**
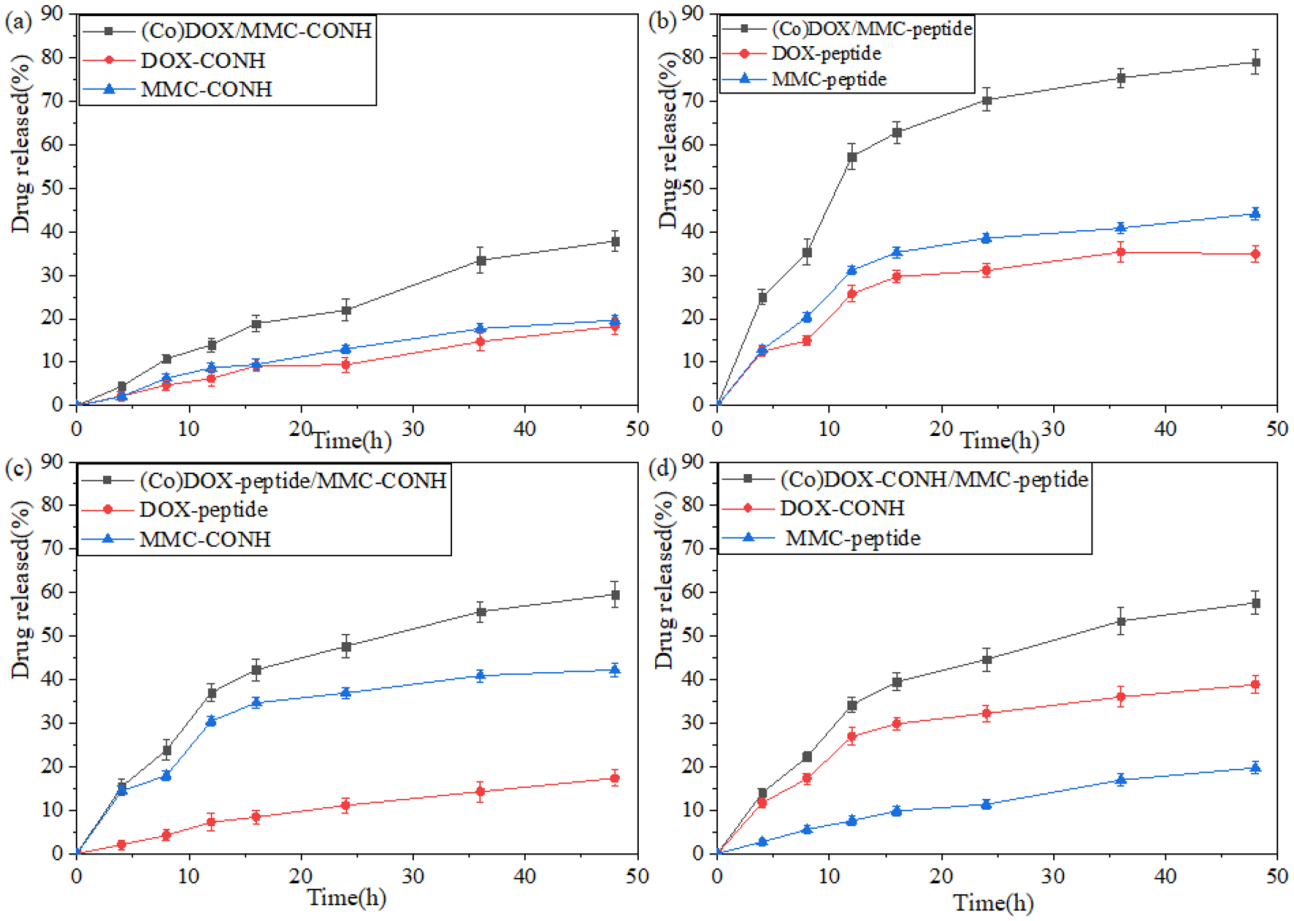
(**a**) Release profiles of (Co)DOX/MMC-CONH, DOX-CONH, MMC-CONH (**b**) Release profiles of (Co)DOX/MMC-peptide, DOX-peptide, MMC-peptide (**c**) Release profiles of (Co)DOX-peptide/MMC-CONH, DOX-peptide, MMC-CONH (**d**) Release profiles of (Co)DOX-CONH/MMC-peptide, DOX-CONH, MMC-peptide. Data were given as mean ± SD (**p < 0.05).

According to previously reports published elsewhere, all highly invasive human tumors exhibit elevated type of collagenase IV activity^19,20^. The in vitro release activities of DOX and MMC from the XG-peptide-MMC/DOX and XG-CONH-MMC/DOX conjugate were tested by culturing the conjugate with collagenase IV at 37 °C. As shown in Fig. 5, DOX and MMC were released as time proceeded. However, when MMC or DOX was conjugated to the polymeric carrier via peptide bond, the drug release was obviously increased compared to the conjugation via amide bond. In these polymer-drug conjugate formulations, the amount of drug released from DOX/MMC-peptide conjugate was found to be approximately 50% at 12 h under processing with collagenase IV and the total release of 73% was reached after 48 h. Compared to other polymer-drug formulations, the total drug release of DOX/MMC-peptide conjugate was remarkably much higher. Hence the drugs were released from the conjugates by the specific hydrolysis of collagenase IV. Therefore, according to this experimental observation, it was noticed that the conjugation of MMC and DOX to the polymeric carrier through peptide bond could significantly be beneficial to achieve the highest and enhanced drug release.

### In vitro cytotoxicity of conjugates against tumor cells

**Figure 6:**
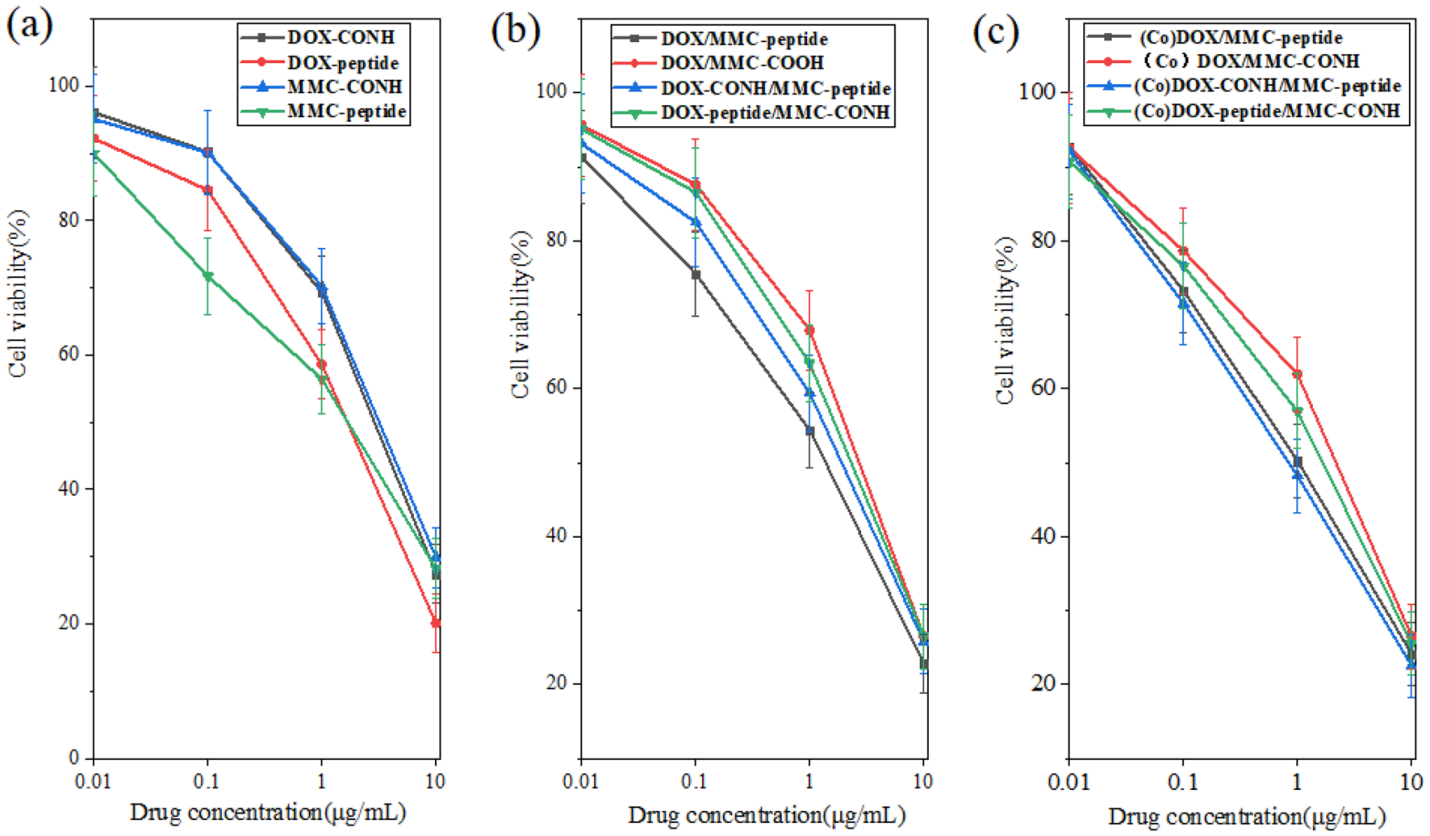
In vitro cytotoxicity of (**a**) DOX-CONH, DOX-peptide, MMC-CONH, MMC-peptide (**b**) DOX/MMC-peptide, DOX/MMC-COOH, DOX-CONH/MMC-peptide, DOX-peptide/MMC-CONH (**c**) (Co)DOX/MMC-peptide, (Co) DOX/MMC-CONH, (Co)DOX-CONH/MMC-peptide, (Co)DOX-peptide/MMC-CONH against the drug resistant HepG2 cells. Data were given as mean ± SD (**p < 0.05).

The in vitro cytotoxicity of different conjugate formulations was investigated against drug resistant HepG2/DR cells by MTT assay. As shown in Fig. 6, MMC-peptide and DOX-peptide showed better effects against cancer cells than MMC-CONH and DOX-CONH, indicating that choosing peptide as spacer in single drug therapies might achieve better therapeutic effects in vitro. In comparison to DOX/MMC-CONH, DOX-CONH/MMC-peptide and DOX-peptide/MMC-CONH, DOX/ MMC-peptide displayed better therapeutic effects against cancer cells. However, the results were different when MMC and DOX conjugated to single XG carrier. In Table 2, the lowest IC_50_ value of 0.85 μg/mL and CI value of 0.49 was detected by treatment with the (Co)DOX-CONH/MMC-peptide formulation, which demonstrated the best cytotoxicity and synergistic effect than other three formulations. It was also shown in Table 2 that when dual drugs were conjugated to single XG carrier the IC_50_ value and CI value became much lower, which indicates that therapeutic effects and synergistic effect were obviously increased relatively to the cocktail mixtures of individual conjugate. Therefore, when dual drugs were conjugated to single XG carrier, the optimal different spacers of DOX and MMC demonstrated more cytotoxicity and synergistic effect than the same linkers.

**Table 2:** IC_50_ and CI values of different formulations in drug resistant HepG2cell (**p <0.05) .

Formulation	IC_50_ μg/mL	CI
DOX-CONH	2.79	
DOX-peptide	1.28	
MMC-CONH	3.24	
MMC-peptide	1.27	
MMC/DOX- peptide	1.05	0.82
MMC/DOX-CONH	2.41	0.80
DOX-CONH/MMC-peptide	1.64	0.94
DOX-peptide/MMC-CONH	2.09	1.14
(Co)MMC/DOX- peptide	1.00	0.78
(Co)MMC/DOX-CONH	1.65	0.55
**(Co)DOX-CONH/MMC-peptide	0.85	0.49
(Co)DOX-peptide/MMC-CONH	1.29	0.70

### Toxicological study

Administration of Gal-XG-MMC conjugate caused no mortality at all doses, indicating that the median lethal dose (LD50) was up to 57.3 mg (MMC eq.)/kg. Compared to the LD50 of MMC that is 13.4 mg/kg, the safety effect of Gal-XG- MMC was significantly improved.

An important indicator of non-specific toxicity after anti-tumor chemotherapy is weight loss. Therefore, we monitored the body weight of the mice after conjugate treatment. The mice treated with the conjugate did not produce any observable side effects, and the weight gain was similar to that of the control group. Their weight increased during the treatment.

The relieving effect of the conjugate on the heart is further supported by the following conclusions: At all doses, there was no significant increase in creatine kinase (CK) or lactate dehydrogenase (LDH) enzyme levels (Table 3). The liver toxicity of the conjugate was evaluated by the serum biochemical parameters and relative liver weight reported in Table 3. Even if the 75 μmol/kg dose was used four times in a row, the conjugate had no significant changes in aspartate aminotransferase (AST), alanine aminotransferase (ALT), LDH and liver weight^9^.

**Table 3:** Serum biochemical parameters and relative liver weight at 2-week after administration of different doses of the conjugates to mice.

Dose (mg/Kg)	ALT (U/L)	AST (U/L)	AST/ALT	LDH (U/L)	CK (U/mL)	Liver/body (w%)
Control	26.7 ± 3.9	32.1 ± 4.5	1.20	483.2 ± 65.3	0.27 ± 0.13	6.27 ± 0.45
MMC/DOX- peptide	27.4 ± 4.8	32.5 ± 4.9	1.19	478.2 ± 56.4	0.33 ± 0.12	6.39 ± 0.46
MMC/DOX-CONH	28.0 ± 4.5	33.2 ± 4.6	1.19	471.1 ± 59.8	0.25 ± 0.11	6.24 ± 0.37
DOX-CONH/MMC-peptide	27.3 ± 3.5	33.7 ± 4.9	1.23	475.6 ± 61.3	0.31 ± 0.18	6.33 ± 0.37
DOX-peptide/MMC-CONH	26.9 ± 4.6	32.4 ± 3.9	1.20	487.2 ± 56.4	0.33 ± 0.29	6.36 ± 0.36

### In vivo antitumor study

**Figure 7:**
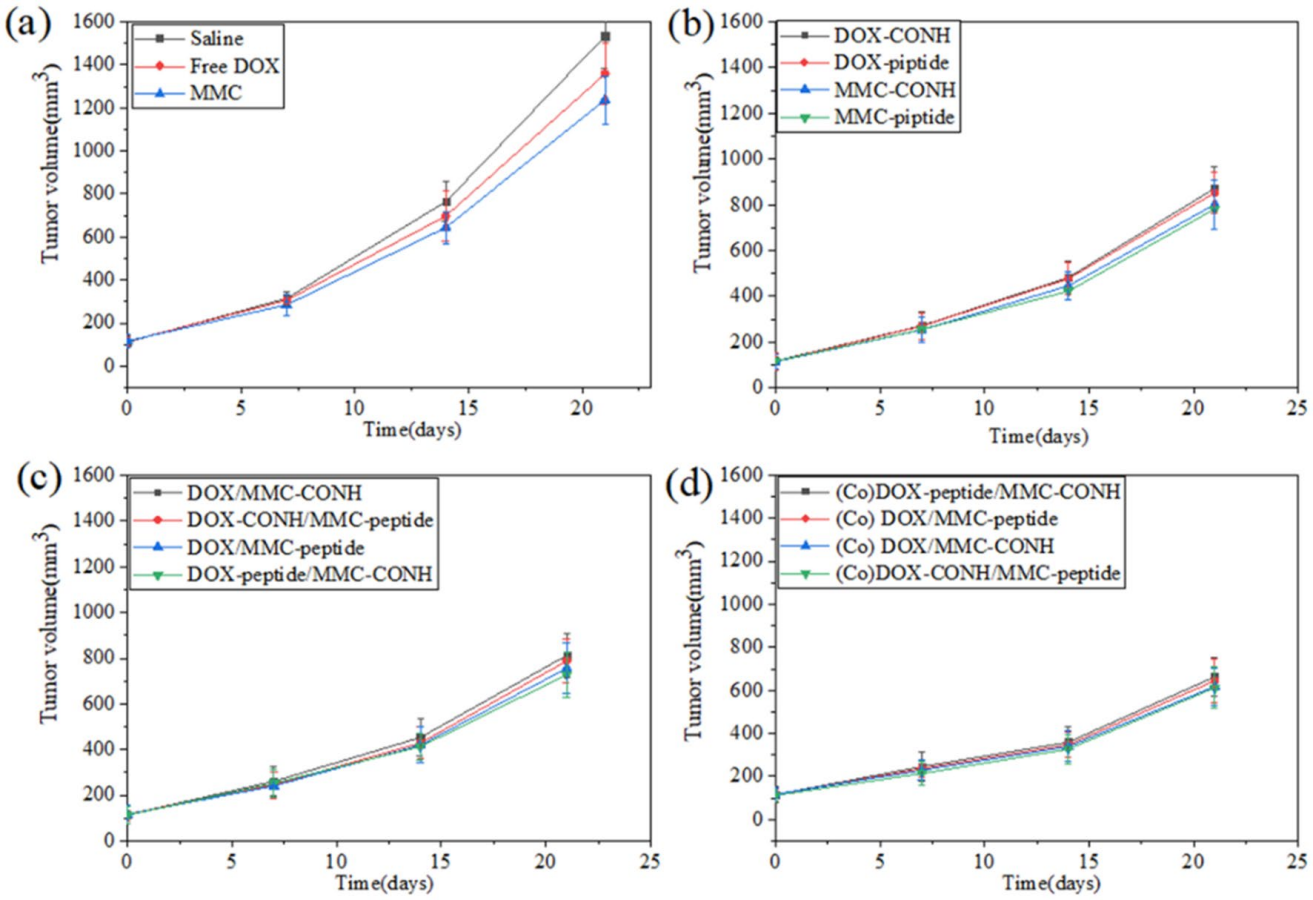
(**a**) In vivo therapeutic efficacy of Saline, free DOX, free MMC (**b**) In vivo therapeutic efficacy of DOX/MMC-CONH, DOX-CONH/MMC-peptide, DOX/MMC-peptide, DOX-peptide/MMC-CONH (**c**) In vivo therapeutic efficacy of (Co) DOX-peptide/MMC-CONH, (Co) DOX/MMC-peptide, (Co) DOX/MMC-CONH, (Co) DOX-CONH/MMC-peptide (**d**) In vivo therapeutic efficacy of DOX-CONH, DOX-peptide, MMC-CONH, MMC-peptide. Tumor size changes of the xenograft nude mice bearing the DOX resistant HepG2 tumors treated with different polymer-drug conjugate formulations. After establishment of HepG2/DR tumor model for 3 weeks into BALB/c nude mice, these DOX resistant HepG2 tumor-bearing mice were treated with drugs (25 μmol/kg) by tail vein injection every week for four doses (days 1, 7, 14, and 21). Data were given as mean ± SD (**p < 0.05).

The in vivo therapeutic efficacy of different conjugate formulations was determined in order to compare their inhibition effects of tumor growth in BALB/c nude mice implanted with drug resistant HepG2/DR cells. The results presented in Fig. 7 showed that compared to free MMC and DOX, polymeric conjugates including DOX-CONH, DOX-peptide, MMC-CONH and MMC-peptide caused distinct fall in the rate of tumor growth. DOX/MMC-CONH, DOX/MMC-peptide, DOX-peptide/MMC-CONH and DOX-CONH/MMC-peptide demonstrated aggressive therapeutic effect against the tumor growth. Especially, when mice were treated with (Co)DOX-CONH/MMC-peptide at a similar dose, the tumor volume growth displayed the slowest rate indicating that (Co)DOX-CONH/MMC-peptide was evidently most effective than other conjugate formulations. These results of in vivo antitumor study showed that when MMC and DOX were conjugated to the single XG carrier through peptide bond for MMC and amide bond for DOX, it could achieve the best effect of tumor inhibition. Therefore, when dual drugs were conjugated to single XG carrier, the optimal different spacers of DOX and MMC achieve ideal synergistic therapeutic efficacy in vivo.

### In Vivo survival rate study

**Figure 8:**
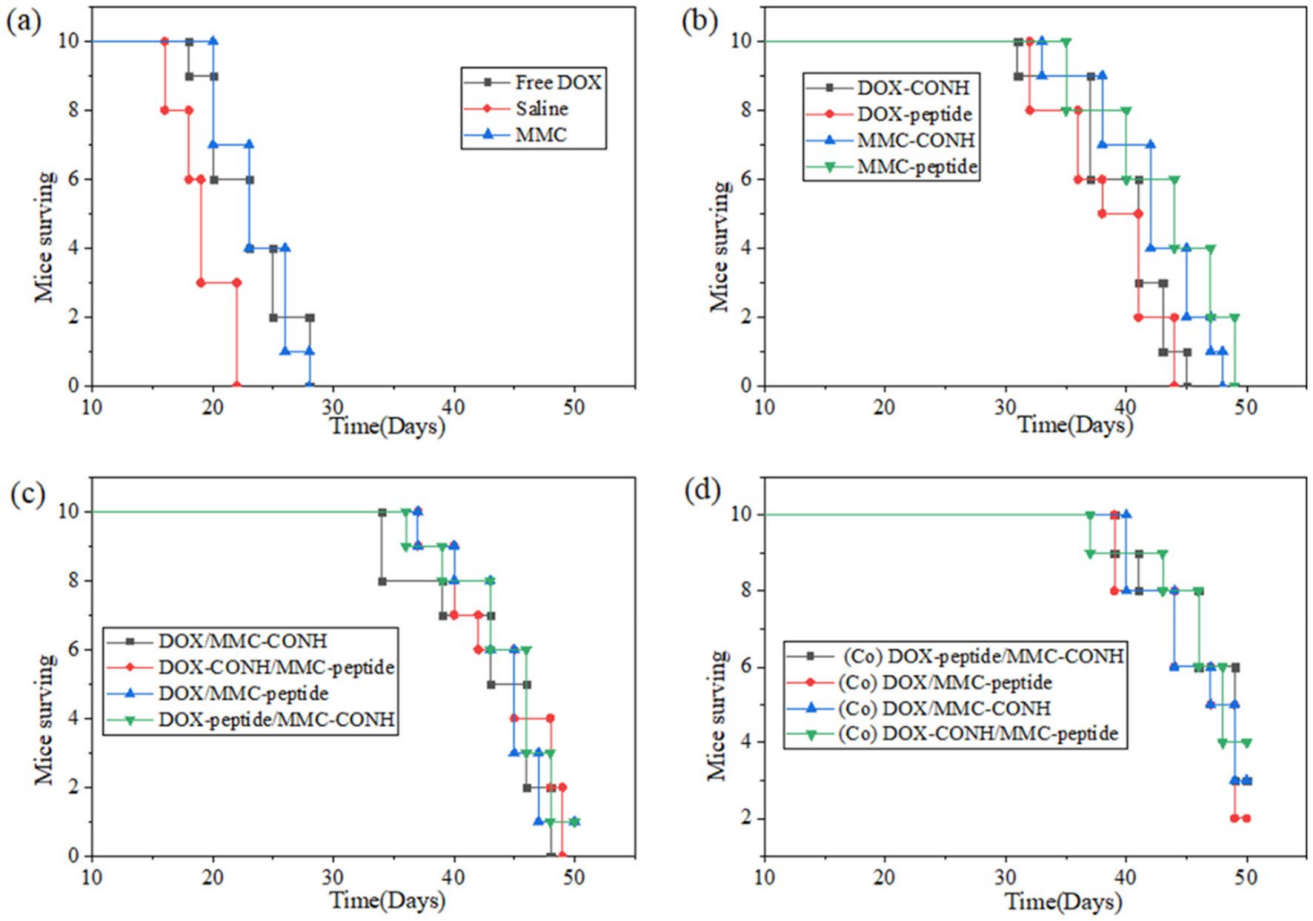
(**a**) Survival curve of the xenograft nude mice bearing the DOX resistant HepG2 tumors treated with free DOX, Saline, free MMC (**b**) Survival curve of the xenograft nude mice bearing the DOX resistant HepG2 tumors treated with DOX-CONH, DOX-peptide, MMC-CONH, MMC-peptide (**c**) survival curve of the xenograft nude mice bearing the DOX resistant HepG2 tumors treated with DOX/MMC-CONH, DOX-CONH/MMC-peptide, DOX/MMC-peptide, DOX-peptide/MMC-CONH (**d**) (Co) DOX-peptide/MMC-CONH, (Co) DOX/MMC-peptide, (Co) DOX/MMC-CONH, (Co) DOX-CONH/MMC-peptide. After growing drug resistant HepG2 tumors for 3 weeks, these DOX resistant HepG2 tumor-bearing mice were treated with drugs (25 μmol/kg) by tail vein injection every week for four doses (days 1, 7, 14, and 21). The survival time and number of long-term survivors (LTS) until day 50 were monitored (**p < 0.05).

The in vivo survival rate of different groups treated with various conjugate formulations was recorded on BALB/c nude mice bearing drug resistant HepG2/DR tumor. The survival data in Fig. 8 showed that the treatment with DOX/MMC-CONH, DOX/MMC-peptide, DOX-peptide/MMC-CONH and MMC-peptide/DOX-CONH displayed increased survival time in comparison with free drug. Administration with (Co)DOX-peptide/MMC-CONH and (Co)MMC-peptide/DOX-CONH produced obviously prolonged survival (46.9 days and 46.8 days, Table 4) compared with administration with slain (19.1 days), free MMC (23.5 days), free DOX (21 days), DOX-peptide (38.5 days), DOX-CONH (39.6 days), MMC-peptide (43 days), MMC-CONH (42 days), DOX/MMC-CONH (42.7 days), DOX/MMC-peptide (44.2 days), MMC-peptide/DOX-CONH (44.3 days) and DOX-peptide/MMC-CONH (44.5 days). Mice treated with the polymer-drug formulations did not show obvious side effects which suggested that that these conjugates may be used to achieve higher therapeutic efficacy. The results showed that the therapeutic and synergistic effect of dual drugs conjugated to single carrier was obviously increased relatively to the cocktail mixtures of individual conjugate.

**Table 4:** Survival time of the xenograft nude mice treated with different polymer-drug conjugate formulations.

Formulation	Survival time (days)
Slain	19.1
Free MMC	23.5
Free DOX	21
DOX-peptide	38.5
MMC-peptide	43
DOX-CONH	39.6
MMC-CONH	42
MMC/DOX- peptide	44.2
MMC/DOX-CONH	42.7
DOX-CONH/MMC-peptide	44.3
DOX-peptide/MMC-CONH	44.5
**(Co)MMC/DOX- peptide	46
**(Co)MMC/DOX-CONH	46.3
**(Co)DOX-CONH/MMC-peptide	46.8
**(Co)DOX-peptide/MMC-CONH	46.9

Data were given as mean ± SD (**p <0.05).

## Discussion

Recently, there has been a great interest in the use of polymer-drug conjugates for drug delivery in combination therapy^21,22^. Conventionally, drugs are attached directly via spacers or bonds to polymeric carriers. Usually, amide or ester bonds are employed, which are sensitive to the pH of tumor tissues or can be hydrolyzed inside the cell by endosomal or lysosomal enzymes^23–26^. There are many enzymes in the lysosomes which have been recognized as important stimulus to achieve efficient intracellular drug release^22,27^. These enzymes can be used to cleave certain peptide. It has been explored that spacer, between drugs and polymer, plays a significant role in controlling drug release. Currently, there are few studies that combine different drugs with different binding bases for combined therapy. Although the macromolecule drug delivery system can deliver drugs simultaneously, the controlled release rate of different binding groups is different, leading to different therapeutic results. Therefore, the application of different spacers for dual drugs in single polymer–drug carrier is expected to improve drug release at the desired site thereby achieving promising therapeutic efficacy. In this study, a series of polymer-drug conjugate formulations of dual drugs (DOX and MMC) were synthesized by amide bond and/or peptide bond to investigate the therapeutic efficacy using different polymer–drug spacers in combination therapy. Tripeptide glycyl-L-leucyl-glycine was chosen as the peptide bond, which could be effectively hydrolyzed by the lysosomal enzymes and to be resistant against attack in the serum^9,28^.

The results from Fig. 2 showed that either for a single drug or dual drugs, drug release could achieve the highest when the bond is peptide. Generally, collagenase IV contains several proteinase components and the specific hydrolysis of collagenase IV for peptide might be stronger than amide bond. This might be the main reason for the higher drug release of DOX-peptide, MMC-peptide and DOX/MMC-peptide conjugate. Moreover, the dosing schedule of MMC and DOX was dependent in drug combination and mechanism of action of the two drugs was different^29^. In addition to in vitro drug release, in vitro cytotoxicity and in vivo cytotoxicity study were measured. The results showed that when DOX and MMC were conjugated to single XG carrier the therapeutic effects and synergistic effect were obviously increased in comparison with the cocktail mixtures of individual conjugate. It was shown in Table 2 that the (Co)DOX-CONH/MMC-peptide formulation had the lowest IC_50_ value of 0.85 μg/mL and CI value of 0.49. Under the same dose and different bonding modes, the release rate of MMC and DOX is different, so that the drug concentration ratio is different. The concentration of DOX is slightly higher than that of MMC, which is beneficial to the inhibition of cancer cells. These might be the reason for the difference in synergistic action of different polymer-drug formations.

The results of in vivo toxicological study also showed that (Co)DOX-CONH/MMC-peptide was most effective than other conjugate formulations (Figs. 5 and 6). In consistency with the in vitro cytotoxicity results, the in vivo cytotoxicity results showed that therapeutic effects and synergistic effect of the dual drugs conjugated to single polymer were obviously increased relatively to the cocktail mixtures of individual conjugate. The survival time of mice treated with the dual drugs in single polymer–drug carrier is two days longer than the life span of mice treated with the dual individual conjugates in Fig. 8. While compared with mice treated with the dual individual conjugates, the tumor volume decreased by 20% in the mice, treated with the dual drugs in single polymer in Fig. 7. The results of in vivo and in vitro therapeutic test were consistent, meanwhile combination index of the dual drugs with single conjugate is about 35% lower than that of the cocktail mixtures of individual conjugate in Table 2. The results indicated that single polymeric carrier carrying dual drugs displayed higher cytotoxicity and synergy than the mixture of the individual conjugate.

The results of in vivo and in vitro toxicity test were consistent; however, these results were inconsistent with the in vitro drug release. Nevertheless, the obtained results about drug release showed inconsistency for in vitro and in vivo experiments. This may be due to the fact that the study of drug release was carried out in buffer incubated with collagenase IV (0.3 mg/mL), however, the cellular environment of tumor cells was complex. There are abundant enzymes including proteases in the lysosomes inside the cell, which play a role in the degradation of drug-polymer spacer to achieve efficient intracellular drug release^23,30,31^. When the polymer-drug conjugates ultimately arrived in the lysosomal compartment of the cell following their pinocytic capture, the degradation of peptide bond and amide bond were different, therefore, the drug release rate was different.

Unlike using same spacer to attach different drugs to polymer vehicle in combination therapy, this research revealed that using different spacer for different drug could achieve better therapeutic effect due to the difference of pharmacological mechanism of different drugs. The results in this study showed that when MMC and DOX were conjugated to the single XG carrier through peptide bond for MMC and amide bond for DOX could achieve best therapeutic effect and synergy effect. Therefore, the outcome of this research could be the indicative of a possibility towards a promising strategy for the optimal design of the polymer-drug conjugates. Controlled release multiple drugs can be used for chemotherapy with different binding bases, to achieve programmable precision treatment of controlled release drugs in accordance with certain program and proportion. The harmony of the spacers for different drugs in single carrier will boost the macromolecular combination therapy for precise medicine.

## Conclusion

Different polymer-drug spacers present diverse therapeutic efficacy so that finding appropriate spacers is desirable especially in combination therapy. This work studied two different spacers, peptide bond and amide bond between XG carrier and anticancer agents (DOX and MMC) and a series of polymer-drug conjugate formulations through different spacers were synthesized. The results showed that the drug release rate became faster when drugs were bonded to the polymer carrier through peptide bond compared with amide bond. The single polymeric carrier carrying dual drugs displayed higher cytotoxicity and synergistic effect than the mixture of the individual conjugate. Using peptide bond for MMC and amide bond for DOX to conjugate dual drugs onto single XG carrier could improve therapeutic effect and synergy effect, that is, there is an optimal design of the polymer-drug conjugates using different spacers. The spacer strategy in polymer-drug conjugates will hold promise and become attractive in drug delivery system for different drug combinations. In the future, the precise chemotherapy needs appropriate harmony linkers to achieve controlled release of multiple drugs in a particular sequence.

## Data Availability

The datasets generated during the current study are not publicly available but are available from the corresponding author on reasonable request.
